# Process evaluation of enhanced community health system activities to improve detection and management of acute malnutrition in Samburu County, Kenya

**DOI:** 10.1371/journal.pgph.0006010

**Published:** 2026-03-24

**Authors:** Monica Nthamba Ng’ang’a, Titus Leokoe, Rita Mukosa, Mbaruka Lekalayo, Samuel Mutua, Teresia Macharia, Mohammed Karama, Tammary Esho, Valerie L. Flax

**Affiliations:** 1 Amref International University, Nairobi, Kenya; 2 Save the Children International, Nairobi, Kenya; 3 Mercy Corps, Nairobi, Kenya; 4 Riccatti College, Nairobi, Kenya; 5 International Rescue Committee, Nairobi, Kenya; 6 RTI International, Durham, North Carolina, United States of America; Washington State University, UNITED STATES OF AMERICA

## Abstract

Acute malnutrition remains a persistent public health concern in Kenya’s arid and semi-arid lands, including Samburu County, where the prevalence of global acute malnutrition (GAM) frequently exceeds the emergency threshold of 15%. Since 2023, a program collaborating with the county government has been implementing a range of enhanced community health systems activities focused on improving early detection and management of acute malnutrition. This process evaluation of the program used qualitative research methods and was guided by the Medical Research Council process evaluation framework. Twenty-five key informant interviews were conducted with program implementers, including 10 community health assistants (CHAs), 10 community health promoters (CHPs), and 5 Samburu County staff. The data were analyzed thematically using Nvivo software. Participants described how training and mentoring on integrated management of acute malnutrition (IMAM) and commodity management improved CHPs’ and CHAs’ knowledge and confidence in acute malnutrition case identification, treatment, and follow-up. Reprinting of paper IMAM registers enhanced monitoring and follow-up of cases, but CHPs had challenges using the new electronic health information system. Most CHAs and Samburu County staff described how the savings and loan associations supported by the project boosted CHPs’ and CHAs’ economic resilience and reinforced their commitment and motivation to do their work. Despite these gains, participants identified persistent barriers to implementation, including logistical and transport challenges, commodity stockouts, limited communications infrastructure, and continued gaps in training and human resources. Misuse of commodities for GAM treatment and community pressure on CHPs, also constrained program implementation. In conclusion, the project should work with the county government to develop strategies to address these challenges and further strengthen program implementation.

## Introduction

Acute malnutrition in children under five represents a major public health concern because it contributes to child morbidity and mortality [1]. Global acute malnutrition (GAM) is prevalent in the arid and semi-arid lands in Africa, where households are affected by shocks and stresses related to recurring and prolonged droughts, among other factors [2]. Samburu County in Kenya is an arid area in the northwest of the country, where the prevalence of GAM frequently exceeds the emergency threshold of 15% [3].

Programs to address GAM are often implemented by community health workers through the community health system (CHS) [4]. Kenya’s community health workers are known as community health promoters (CHPs), and they play a pivotal role as the initial touchpoint for community members seeking health-related services, including health education and fundamental curative health services within the community [5]. A multisectoral program in Samburu and Turkana Counties to address the persistent problem of GAM conducted a formative study to refine and adapt its CHS support component. The study found that CHPs who received supervision from community health assistants (CHAs) were able to identify acute malnutrition cases, follow-up children who did not complete treatment for acute malnutrition, and facilitate community engagement activities, like action days and dialogue forums [6]. The study also highlighted significant gaps related to CHP training; CHPs’ use of certain supplies and commodities; stockouts of supplies and commodities; and community mistrust of CHPs. Additionally, delays in payment of CHPs’ monthly stipends by the county government or complete nonpayment of stipends negatively affected CHP morale and motivation, further undermining their efforts to address acute malnutrition [6].

Using these initial findings to guide design, the program began collaborating with the counties to implement enhanced CHS activities in 2023. Throughout implementation, the program has collected regular monitoring data to understand the number of people exposed to or engaged by the program activities. However, data about the process of implementation from the perspective of the implementers has been lacking. Process evaluations are useful for programs because they assess how well activities are being implemented and whether implementation is proceeding as planned [7]. This study contributes to filling this gap by conducting a process evaluation of the program’s enhanced CHS activities on acute malnutrition reduction in Samburu County with the aim of uncovering factors that contribute to the success of implementation and practical and contextual challenges to implementation.

## Methods

### Study design and setting

This research is a qualitative process evaluation of the program’s CHS activities in Samburu County. It was guided by the Medical Research Council (MRC) process evaluation framework to examine how, why, and under what circumstances the program’s enhanced CHS activities could lead to improved acute malnutrition outcomes [8].

This study was conducted in Samburu County, where the majority ethnic group is Samburu and other ethnic groups include Turkana, Rendille, Borana, and Pokot. The county has three livelihood zones: pastoral, agro-pastoral, and urban/peri-urban. One hundred and twenty out of 140 community units (CUs) in Samburu County are active. As the first level of the health system, CUs promote healthy practices, identify cases for referral, and support community-level management of illnesses. Each CU covers about 5,000 people, with one CHP for every 25 households, supervised by a CHA overseeing 25 CHPs [9].

### Eligibility

Participants were eligible for this research if they were working as CHAs, CHPs, or Samburu County staff; were 18 years or older; and willing to participate in the study and provide informed consent.

### Sample and sampling procedures

Data were collected from 13 CUs in Samburu County, specifically: Morijo, Longewan, Naiborkeju, Porro, Loosuk, Lodokejek, Loltulelei, Seketet, Loiboingare, Lmisigigiyoi, Kisima, Nkejemuny, and Sirata. These CUs were purposefully selected to ensure a broad representation of different geographic locations and community contexts within the project area. From these units, 25 key informants were identified based on their roles, experience, and involvement in the program’s CHS-related activities. The sample included 10 CHAs, 10 CHPs, and 5 Samburu County staff. We sampled one CHA and one CHP per CU and included them in the study depending on availability.

The key informants were chosen from a larger pool of potential participants identified through purposive sampling. Selection was guided by their relevance to the study objectives, professional roles, and level of involvement in the program. This approach ensured that informants had firsthand knowledge and experience necessary to provide rich, context-specific insights into the study topic. The qualitative sample sizes were selected to achieve saturation in the overall sample. Saturation is the point in qualitative research at which no new information is obtained through additional data collection [10]. Data saturation was evaluated during data collection and preliminary analysis for each respondent group and considered reached once no new themes emerged.

### Data collection and reflexivity

The key informant interviews (KII) question guides were based on elements of the MRC process evaluation framework. They were developed in English and translated into Samburu and Kiswahili. KIIs were conducted by a trained research assistant from the county and fluent in the local language. The main information solicited from the KIIs focused on enhanced CHS activities related to acute malnutrition reduction, factors contributing to the success of those activities, and challenges/barriers to implementation.

Data were collected from April 7^th^ to April 17^th^ 2025. The KIIs were conducted in private rooms, at the CHAs’ offices, or through audio calls. KIIs with 10 CHPs and five CHAs were conducted via audio calls while KIIs with five CHAs and all five Samburu County staff were conducted physically at their offices. The interviews were digitally recorded and conducted in English, Kiswahili, or the Samburu language based on study participants’ preferences. The recorded interviews were transcribed by a trained transcriber. Recordings in Kiswahili or Samburu were transcribed directly into English. The initial transcripts were thoroughly reviewed to ensure that data quality was good and feedback was provided to the research assistant as needed.

The research team included public health and social science researchers experienced in Kenya’s CHS and nutrition programs. While some had prior professional familiarity with the implementing organization and intervention model, they were not involved in supervision of CHPs and CHAs, which could have influenced interpretation. To mitigate potential bias, an independent researcher collected data using tools that examined both successes and challenges, and analytical rigor was enhanced by reporting both to strengthen transparency and credibility.

### Data analysis

The data were analyzed by MN and SM with Nvivo software (version 15, Lumivero, Denver, Colorado), using thematic content analysis methods [11]. Codebooks were developed for each type of participant using deductive codes based on the question guide and MRC framework. Inductive codes were added during the coding process. Relevant codes were grouped into key themes and code reports were used to create summaries for each theme.

### Ethical considerations

Ethical approval was obtained from AMREF AMIU/ ARP/ 5813–1 and ESRC P1835-2025. All participants provided written consent. Participation was voluntary, and no coercion was used. The risks and benefits of the study were explained to all participants. No incentive was given to the study participants.

## Results

The themes are drawn from the MRC process evaluation framework and key interventions implemented by the program under the CHS component.

### Intervention and implementation components

Table 1 describes the intervention activities, how they are implemented, and the motivation for each activity. The program implements all interventions through the county government CHS in collaboration with the county health team.

**Table 1:** Enhanced CHS activities implemented.

Activity	How the programs support the activity	Reason/ Motivation for the activity
Support nutrition technical forums	Provides financial and technical support for nutrition technical forums at county and sub-county levels. Participants include county department of health staff and implementing partners.	To address gaps, strengthen advocacy, enhance layering of services, and improve service delivery.
Mentorship and training on commodity management	Supports the counties to train and carry out routine mentorship of CHPs and CHAs on commodity management, including training on quantification and forecasting of health and nutrition commodities.	Health facilities were not adequately forecasting, ordering, and supplying commodities, such as ready-to-use-therapeutic foods (RUTFs), ready-to-use supplementary foods (RUSFs), and did not account for commodities CHPs needed.
Integrated health and nutrition mobile outreach	Facilitates integrated health and nutrition outreach activities in communities to: mobilize community members and sensitize them on health seeking behaviors, childcare and feeding practices, child immunization, hand washing practices, safe management of household drinking water, and child feces disposal practices. offer these services: growth monitoring, GAM assessment, diagnosis and treatment of minor illness. refer those who need advanced assessments and management.	To bridge the challenge communities face in physical access to nutrition and preventive health services by offering them in the community.
Integrated management of acute malnutrition (IMAM) training and mentorship and reprinting of IMAM registers	IMAM is a community and facility-based approach to managing acute malnutrition. Children with acute malnutrition without medical complications are treated as outpatients. Children with acute malnutrition and medical complications are treated as inpatients [12]. The program supports training of CHPs and CHAs on IMAM by certified MoH trainers of trainers and printing of IMAM registers.	Training was supported to improve the health system’s sensitivity and responsiveness to increased acute malnutrition caseloads at community and facility levels. Reprinting of IMAM registers was supported to create reliable data for early detection and response to increases in cases of acute malnutrition.
Community health unit savings and loan association (CHUSLA) training	CHUSLA is a savings group that pools resources to provide members with access to savings and credit. These associations are particularly common in low-income and rural areas where formal banking services are limited [13]. The program supports training of CHPs and CHAs on CHUSLA. The training follows the voluntary savings and loans associations (VSLA) curriculum in consultation with the Ministry of Trade, Industry, and Cooperatives. The groups are provided with tool kits, which include savings boxes and record books.	To motivate CHPs as a financial incentive and protect CHPs and their families from shocks as they continue to deliver health and nutrition services in the community.
Linkages to nutrition resilience interventions	Creates linkages with nutrition-sensitive activities, such as VSLAs among mother-to-mother support groups.	VSLAs enable women in the community to access finance and affordable credit, which helps them to meet household nutritional needs.

### Implementation process and implementers’ experience with the intervention

In this section, we describe the implementation process and implementers’ experience with each intervention component, including successes and ongoing challenges to implementation. Responses from CHPs, CHAs, and county government representatives were similar across types of participants, reflecting alignment in how they understood and experienced the intervention.

### Theme 1: Mentorship and training on commodity management

Availability and management of commodities is critical to the delivery of health and nutrition services, particularly for managing acute malnutrition. The program provides CHPs and CHAs with mid-upper arm circumference (MUAC) tapes for malnutrition screening and thermometers and respiratory timers for identification of common illnesses. At the outset, the program found that CHPs had limited knowledge on utilization of these commodities and stockouts of commodities, such as ready-to-use therapeutic food (RUTF) and ready-to-use supplementary food (RUSF), were common in some health facilities. To address this, the program trained and provided routine mentorship on commodity management to CHPs, health facility nurses, and CHAs. This was intended to enable CHPs to perform key roles including forecasting, ordering, accountability, pharmacovigilance, and reporting. CHPs, CHAs, and county government staff shared the following insights on the improvements and ongoing challenges in commodity management.

### Timely procurement and increased commodity demand

According to most county health staff, training enabled CHPs and CHAs to request commodities in a timely manner, reducing delays. A county staff member explained, “*Because of training, CHAs and CHPs can request the commodities in time.*” Due to good uptake of commodities, some health facilities experienced stockouts, reflecting a growing demand for commodities, such as RUTF and RUSF. A county government staff member explained, “*Because of good uptake of the commodities, we have experienced stockouts*.” Another challenge was lack of proper storage facilities for commodities, such as RUTF and RUSF. This led them to be stored with other medications, which can result in mix-ups or spoilage. A CHA explained, “*We lack storage for the RUTF and RUSF in our dispensary which forces us to store them with other medications*.”

### Perceived improved knowledge and skill in commodity management

Most CHPs and CHAs said that the training they had received helped them gain an understanding of how to give out commodities such as paracetamol, deworming medication, RUSF, and RUTF. A CHA highlighted this by saying, *“The training has been helpful in improving our skills in commodity management. We now know how to properly record the distribution of commodities, identify their expiry dates, and determine the correct quantity a child should receive based on their weight.”*

### Strengthened service delivery

Most CHPs and CHAs said that they learned during training how to use MUAC tapes to screen children for acute malnutrition and provide appropriate nutritional supplements. A CHA explained, “*We screen for malnutrition using MUAC tapes, and if the child is identified within the yellow or red categories, they are provided with the appropriate nutritional supplements such as RUSF or RUTF*.”

### Enhanced community engagement and awareness

According to most CHPs and CHAs, due to the training, they are able to educate community members on the importance and proper use of nutrition commodities, such as RUTF and RUSF, referred to below as Plumpy’Nut. CHPs and CHAs raise awareness on misuse of commodities, particularly around inappropriate distribution to healthy children. A CHP explained, “*When Plumpy’Nut is distributed, it is meant for children who have been diagnosed with malnutrition. However, some parents also give it to healthy children. Because of the training we received, we had to step in and raise awareness in the community, explaining that giving Plumpy’Nut to children who do not need it can be harmful [because the child who has acute malnutrition will* *not improve]*.” Some CHPs mentioned that some community members believe that CHPs and CHAs use favoritism when distributing nutrition commodities and this can lead to resentment within the community.

### Better monitoring and reporting

Some CHAs who have smartphones reported that they have improved the accuracy of their reporting and household-level tracking of commodities, such as Plumpy’Nut. The introduction of bin cards (commodity management recording/documentation tools to record incoming and outgoing stock movement) at health facilities has helped monitor the distribution of commodities to CHPs. According to a CHA, “*We have bin cards which we use to record when issuing commodities to CHPs*.”

### Theme 2: IMAM training and mentorship

The program trained CHPs and CHAs on prevention, identification, and treatment/management of acute malnutrition. This section describes improvements and ongoing challenges observed by participants following training and mentoring.

### Perceived changes in length of stay and cure

Most CHPs, CHAs, and county government staff reported that due to training and mentorship, some malnourished children recover within a short period. A CHA explained, “*A malnourished child recovered within two weeks after being put on appropriate commodities and follow-up.”* Children who received commodities such as RUTF and RUSF were described as showing significant improvements in their health. County government staff attributed this to availability of the nutrition commodities in the right quantities.

### Perceived changes in malnutrition and relapse cases

Most CHPs, CHAs, and county government staff reported that the number of malnourished children has decreased. They attributed this to early detection and consistent treatment of acute malnutrition cases. A CHP explained, “*There is improvement because when we do screening, we detect malnutrition early and refer them to health centers. They get the RUSF and/or RUTF at the right time and malnutrition is managed before it becomes severe.*” Training parents and caregivers on nutrition, kitchen gardening, and balanced diets was described as contributing to improved household dietary practices. A CHP explained, “*Because of increased uptake of vegetables, the malnutrition cases have reduced*.” Additionally, CHPs in agropastoral and urban/peri-urban livelihoods said there are fewer relapse cases because they follow-up with clients and refer defaulters back to the health facilities where they are re-admitted to the program to ensure continuity of treatment. However, among pastoralists default remained high because of migration in search of pasture for livestock.

### Theme 3: Reprinting and use of IMAM registers

The program reprinted and supported the use of IMAM registers with the aim of creating robust and reliable data for early detection and response. This section describes improvements and challenges related to IMAM registers mentioned by the participants.

### Perceived changes in documentation

Most county government staff said that availability of IMAM registers has enhanced documentation at the primary level, thereby strengthening overall data quality. A county government staff member said, “*It helped in terms of documentation because when you have the right tools, documentation improves. We were able to capture data and when data is captured at the primary level, the quality of the reports is strengthened.”* According to some county government staff, they reported that improved data quality also supports evidence-based decision making for nutrition interventions. In addition, CHPs are now better equipped to trace non-compliance and follow up with patients who miss appointments, enhancing treatment continuity. The registers enabled nutritionists to calculate treatment outcomes and key indicators, such as cure and relapse rates. In addition, they helped in identifying relapse cases and geographic areas where malnutrition remains a concern.

### ICT infrastructure challenges

Most county government staff reported that the electronic Community Health Information System (eCHIS) rolled out at the community level was not well understood by most CHPs. A county government staff member explained, “*We have a problem with the eCHIS system that is currently being deployed at the CU level. Many CHPs and CHAs have not understood it or how to navigate through the system, which is used to report service delivery and administration*.”

### Theme 4: Integrated health and nutrition outreach

The program organizes health and nutrition outreach sessions to deliver quality health and nutrition services closer to communities where health facilities are not available. This makes it possible to detect disease and acute malnutrition cases early and to provide information and education that can influence nutrition and health-seeking behaviors. Participants reported the following improvements and challenges they observed.

### Perceived changes in access to services

According to most CHPs, CHAs, and county government staff, the outreach sessions have been instrumental in reaching people in remote areas and reducing distance barriers to receiving basic health services. A CHP said, “*It helps because I can reach people who live far away and enable them to access health services closer to their homesteads, hence breaking the distance barrier*.” The outreach sessions have enabled screening, detection of malnutrition, and referral for treatment as well as diagnosis and treatment of morbidities causing malnutrition. A CHP explained, “*It has helped me because I can screen many children on my own*.” Despite this improvement, most CHPs and CHAs mentioned there were still logistical and geographical barriers, especially during the rainy seasons when mobility is hampered causing delays or cancellation of planned outreach activities. A CHP reported, “*We experience poor infrastructure on the roads, especially when there are heavy rains*.”

### Enhanced health facility and community linkage

Most CHPs, CHAs, and county government staff reported that the outreach sessions have built trust and stronger relationships between CHPs and the community. A CHA stated, “*It helps build trust and good relationships between the community and CHPs because of the services we provide.”* Despite this improvement, according to some CHPs and CHAs, some community members still believed CHPs benefit financially from outreach activities. A CHP explained, “*The community believes we benefit from the outreaches, while they don’t get anything financially. They think we use them for financial gain.*”

### Theme 5: Community Health Unit Savings and Loan Association (CHUSLA) training

At the beginning of the program, there were delays in government payment of stipends or complete non-payment, which undermined CHPs’ morale and motivation to carry out activities related to acute malnutrition. To mitigate this, the program trained CHPs and CHAs on CHUSLA implementation to boost their motivation and provide social protection as they delivered health and nutrition services in the community.

According to most CHPs, CHAs, and county government staff, CHUSLA training and CHPs’ participation in CHUSLA helped them to improve financial management and savings culture, enhance entrepreneurship, and build assets. This enabled them to accumulate funds for personal or family needs. As mentioned by a county government staff member, “*Now CHPs have money. They don’t depend only on the stipend they get from the government but also on [the money they make in the] other businesses they have started as a result of CHUSLA savings.”* The program has enabled the establishment of well-structured savings groups with loan systems. According to a county government staff member, “*Training CHPs was highly impactful and significant, as many have applied the skills gained from CHUSLA to improve their livelihoods. Some have joined table banking groups (savings group where members come together to pool their savings and provide loans to one another), started small businesses, or become active members of community organizations to support themselves sustainably.”*

The challenges experienced with CHUSLA were financial constraints, such as CHPs’ lacking money to make regular contributions. In some cases, participants described the payout as insufficient for CHPs’ expenses. A CHP explained, “*The money that we share at the end of the month is not enough to cater for everything.”* Some other issues include low attendance by members, and long travel distances for some CHPs to participate in the CHUSLA groups. As explained by one CHP, “*Some [CHPs] don’t come to meetings because of long distances and they have no phone to reach them.”* Limited literacy skills among CHPs also leads to poor financial record keeping and unequal contributions by CHPs cause tension among members. As one CHP explained, “*Due to varying contribution amounts, where some give more than others, a few members feel discouraged and consider leaving*.”

## Discussion

This study evaluated the implementation of enhanced CHS activities targeted at acute malnutrition reduction in Samburu County, Kenya. Participants described several successes in implementation, including more timely procurement and management of commodities, better referral and follow-up through IMAM training, enhanced documentation of acute malnutrition cases via IMAM registers, and increased community access to health-related services through integrated outreach. However, challenges such as stockouts, limited storage, shortages of trained personnel, financial constraints, logistical barriers, and community mistrust were still persistent.

Findings from this study revealed that CHPs, CHAs, and county staff felt that mentorship and training for CHPs on IMAM helped CHPs improve their detection and follow up of acute malnutrition cases, while training on commodity management improved community level access to health commodities. Previous programs in Kenya’s arid and semi-arid lands showed that training CHPs on the IMAM approach enabled successful mobilization of human resources and supplies for identifying and treating children with acute malnutrition at times when cases spike [14]. In this project, the increases in detection and treatment of acute malnutrition cases that occurred after CHPs were trained led some health facilities to experience occasional stockouts, indicating rising demand that outpaced current supply capacity. This underscores the need for improved supply chain responsiveness and forecasting mechanisms. Maintaining regular access to malnutrition treatment services and supplies is crucial for sustaining community trust and service utilization [13]. In a project in South Sudan, health workers reported that stock shortages and supply interruptions negatively influenced community trust in the intervention, leading to decreased attendance and service uptake [4]. Repeated stockouts of RUTF in a program in Kenya and Ethiopia led communities to view acute malnutrition treatment services as unreliable, resulting in longer treatment durations, increased absenteeism, and higher non-compliance rates [13]. The program in Ethiopia addressed this challenge by implementing a communication system between providers and caregivers to ensure that changes in the availability of RUTF were clearly conveyed, which reduced non-compliance during periods of stockouts [13]. The present project in Samburu County may want to adopt a similar type of system and adapt it for this context. To address these challenges, Puett et al. suggested implementing a well-structured communication system between providers and caregivers [13]. This approach helped ensure that changes in the availability of RUTF were clearly conveyed, which in turn reduced non-compliance rates during periods of stockouts [13].

This study found that the reprinting and distribution of physical IMAM registers was helpful to CHPs and CHAs for tracking children with acute malnutrition, but CHPs were not successful at using the eCHIS. This was likely related both to the low level of literacy of most CHPs and lack of training and support on eCHIS use. A recent review in low-and middle-income countries found that low technical capacity and existence of parallel reporting tools (e.g., paper and electronic) are barriers to effective implementation of community health information systems in these settings [15]. The program should work with the county government to streamline parallel reporting systems, reinforce training and support for CHPs on the use of eCHIS, and continue strengthening supply forecasting and quantification systems through eCHIS to accurately reflect demand.

Participants in this study felt that integrated health and nutrition outreach improved access to services in remote areas, thereby increasing service coverage, enhancing health facility and community linkage, and enabling early detection and follow-up of acute malnutrition. Findings from this study found that long distances, lack of transport and low turnout affected the success of outreach services in some cases. A review of community health worker programs in low-and middle-income countries found that community health workers frequently lacked transportation for themselves and their clients, and this impacted their performance [16]. Providing community health workers or CHPs with transport support, whether financial assistance or vehicles like bicycles or motorbikes, helps them reach remote and underserved populations [17]. For example, a program in Angola, addressed RUTF distribution challenges by equipping community health workers with bicycles. These were later replaced with locally hired three-wheeled motorbikes, which worked better for transporting larger quantities of RUTF across difficult terrain. The program should adapt transport solutions so that CHPs can more easily conduct outreach in remote and underserved areas.

Participants in this study said that CHUSLA training enabled CHPs to save money, enhance their entrepreneurship, and establish well-structured savings groups with loan systems. Substantial evidence shows that involvement in savings groups leads to increased savings and credit use and also contributes to improved food security, resilience, self-confidence, and a stronger sense of solidarity among members [18]. This is especially important for CHPs in Samburu County because their government stipend is low and not always provided on a regular schedule, so participation in savings groups can help them set up alternative livelihood activities, while also allowing them to keep working as CHPs. The program should continue supporting the formalization of group structures and develop standardized guidelines on recordkeeping, group rules, conflict management, and savings/loan disbursement processes.

### Study strengths and limitations

This study had several strengths. We collected data from a diverse range of stakeholders, providing a broad perspective on the implementation of an enhanced CHS intervention, including improvements in skills, service uptake, and socio-economic empowerment through CHUSLA. It offered a holistic view by capturing multiple dimensions of community health work, such as training, service delivery, and systems strengthening, validated through firsthand accounts from CHPs, CHAs, and county government staff. The study also had some limitations. It was conducted in one county, which may affect the transferability of its findings. However, learnings from this study could be applied in other arid or semi-arid lands or pastoral/agropastoral contexts in Kenya, such as Turkana, Marsabit, and Isiolo Counties, with similar seasonal adverse climatic conditions and high acute malnutrition prevalence.

## Conclusion

This study found that the implementation of enhanced CHS activities in collaboration with the county government in Samburu County strengthened CHPs’ and CHAs’ skills to identify, treat, and follow-up acute malnutrition cases. However, to sustain and scale these gains, it is essential to address persistent challenges such as frequent stockouts, weak supply chains, limited ICT infrastructure and understanding of electronic health system use, and social and logistical barriers. The county government in collaboration with the project and other development partners should use the findings from this evaluation to further adapt the enhanced strategies to address these challenges and improve the long-term resilience of CHS activities to identify and treat acute malnutrition.

## Supporting information

S1 Table Results and Summaries of In-depth Interviews with Samburu County Staff. (DOCX)

S2 Table Results and Summaries of In-depth Interviews with Community Health Assistants and Community Health Promoters. (DOCX)

S1 File Inclusivity in global research. (DOCX)

## References

[1] Schwinger C, Golden MH, Grellety E, Roberfroid D, Guesdon B. Severe acute malnutrition and mortality in children in the community: Comparison of indicators in a multi-country pooled analysis. PLoS One. 2019;14(8):e0219745. doi: 10.1371/journal.pone.0219745 31386678 PMC6684062

[2] Young H, Marshak A. Persistent Global Acute Malnutrition: A discussion paper on the scope of the problem, its drivers, and recommendations for policy, practice, and research. Feinstein International Center, Tufts University. 2017. https://fic.tufts.edu/wp-content/uploads/FIC-Publication-Persistent-Global-Acute-Malnutrition_web_2.26s.pdf

[3] Ministry of Health (Kenya). Kenya Nutrition Situation Overview. Nairobi: Ministry of Health. 2024.

[4] López-Ejeda N, Charle Cuellar P, Vargas A, Guerrero S. Can community health workers manage uncomplicated severe acute malnutrition? A review of operational experiences in delivering severe acute malnutrition treatment through community health platforms. Matern Child Nutr. 2019;15(2):e12719. doi: 10.1111/mcn.12719 30315743 PMC6587873

[5] Ministry of Health (Kenya). Kenya Community Health Strategy 2020-2025. Nairobi: Ministry of Health. 2021. http://guidelines.health.go.ke/#/category/12/447/meta

[6] Ng’ang’a MN, Thuita F, Ngari M, Webale A, Macharia T, Eris S, et al. Factors that influence acute malnutrition detection and treatment by community health promoters in Samburu and Turkana counties, Kenya: A mixed methods study. PLOS Glob Public Health. 2026;6(1):e0005689. doi: 10.1371/journal.pgph.0005689 41563981 PMC12822924

[7] Menon P, Covic NM, Harrigan PB, Horton SE, Kazi NM, Lamstein S, et al. Strengthening implementation and utilization of nutrition interventions through research: a framework and research agenda. Ann N Y Acad Sci. 2014;1332:39–59. doi: 10.1111/nyas.12447 24934307

[8] Moore GF, Audrey S, Barker M, Bond L, Bonell C, Hardeman W, et al. Process evaluation of complex interventions: Medical Research Council guidance. BMJ. 2015;350:h1258. doi: 10.1136/bmj.h1258 25791983 PMC4366184

[9] Ministry of Health. Kenya Health Policy 2014-2030. Nairobi: Ministry of Health. 2014. https://extranet.who.int/countryplanningcycles/planning-cycle-files/kenya-kenya-health-policy-2014-2030

[10] Bernard HR. Research Methods in Anthropology: Qualitative and Quantitative Approaches. 4th ed. Lanham, Maryland: AltaMira Press/Rowman & Littlefield. 2006.

[11] Braun V, Clarke V. Thematic analysis: A practical guide. London: SAGE Publications Ltd. 2022.

[12] Ministry of Medical Services, Ministry of Public Health and Sanitation. National Guideline for Integrated Management of Acute Malnutrition. Nairobi: Ministry of Health; 2009. Available from: http://guidelines.health.go.ke:8000/media/IMAM_Guideline_Kenya_June09.pdf

[13] Puett C, Swan H, Guerrero S. Access for All, Volume 2: What factors influence access to community-based treatment of severe acute malnutrition? London: Coverage Monitoring Network. 2013. https://www.coverage-monitoring.org/wp-content/uploads/2013/11/Access-for-All-Volume-2.pdf

[14] Ngetich W, Gichohi G, Wambua F, Daniel T, Yishak Y, Codjia P. Implementing the IMAM Surge approach-experiences from Kenya. Field Exchange. 2021;64:22–6.

[15] Mekonnen ZA, Chanyalew MA, Tilahun B, Gullslett MK, Mengiste SA. Lessons and Implementation Challenges of Community Health Information System in LMICs: A Scoping Review of Literature. Online J Public Health Inform. 2022;14(1):e5. doi: 10.5210/ojphi.v14i1.12731 36457350 PMC9699826

[16] Kok MC, Dieleman M, Taegtmeyer M, Broerse JEW, Kane SS, Ormel H, et al. Which intervention design factors influence performance of community health workers in low- and middle-income countries? A systematic review. Health Policy Plan. 2015;30(9):1207–27. doi: 10.1093/heapol/czu126 25500559 PMC4597042

[17] Agarwal S, Sripad P, Johnson C, Kirk K, Bellows B, Ana J, et al. A conceptual framework for measuring community health workforce performance within primary health care systems. Hum Resour Health. 2019;17(1):86. doi: 10.1186/s12960-019-0422-0 31747947 PMC6868857

[18] Gash M. Understanding the impact of savings groups. Financial Sector Deepening Africa: SEEP Network. 2017. https://www.rfilc.org/wp-content/uploads/2020/08/SEEP_Understanding-the-Impact-of-Savings-Groups_20180117_FINAL.pdf

