# Supplementary material for: Process evaluation of enhanced community health system activities to improve detection and management of acute malnutrition in Samburu County, Kenya
Source: PLOS Glob Public Health. 2026 Mar 24;6(3):e0006010. doi: 10.1371/journal.pgph.0006010 (PMC13012446; doi:10.1371/journal.pgph.0006010)
Supplement: S1 Table — (DOCX) [file pgph.0006010.s001.docx]

**S1 Table. Results and Summaries of In-depth Interviews with Samburu County Staff**

| **Questions** | **Responses** | **Summaries** |
| --- | --- | --- |
| **Routine mentorship and training of HCWs on commodity management** | | |
| 1. **How has the training and mentorship on commodity management improved the ability of HCWs to manage and distribute essential supplies?** | - I don’t think there is specific commodity management training that has happened in my department. - We still have gaps in terms of how to manage commodities from paperwork up to usage by CHAs and CHPs. - There is an impact because before HCWs were unable to write IMAM reports (713 reporting tool). They have improved. - Before, they left commodity management to CHPs but now they are involved because of training. - In terms of storage, before they were storing IMAM commodities together with medicine, but after training every facility has allocated space for IMAM commodities. - Because of training, there are no losses. - Also, they are able to make correct diagnosis and interventions. | 1. **Improved reporting**: HCWs are now able to complete IMAM reports e.g., the MOH 513 reporting tool which they previously struggled with. 2. **Increased involvement**: HCWs are now actively involved in commodity management, a task previously left to CHPs. 3. **Better storage practices**: facilities have allocated specific storage areas for IMAM commodities instead of mixing them with general medicines. 4. **Reduced losses**: training has contributed to minimizing losses of commodities. 5. **Improved diagnosis and intervention**: training has helped HCWs make correct diagnoses and apply appropriate interventions. |
| 1. **What changes have you noticed in commodity availability or reductions in stockouts since these activities began?** | - We have noticed changes because currently where we have CHPs with commodities, we are able to support those individuals at household level then we can refer them where it is necessary. We manage commodities at CHP level. - Yes, because of good uptake of the commodities, we have experienced stockouts. - Because of training, HCWs can procure those commodities in time. - Short expiry date commodities which mean cannot be absorbed in the facility. | 1. **Improved community-level access**: CHPs now manage commodities at household level, supporting individuals directly before referring them if necessary. 2. **Timely procurement**: Training has enabled HCWs to procure commodities in a timely manner, reducing delays. 3. **Increased demand**: due to good uptake of commodities, some facilities have experienced stockouts, indicating higher utilization. |
| 1. **What challenges, if any, remain in ensuring adequate commodity management across facilities?** | - We have challenges and one is to get tool to support CHPs to understand the medication of each commodity. - The challenge of accessing the commodities/ procurement of these commodities in the hierarchy - We need to quantify the commodities that we use so that it can help us procure what we require not the current system where we are being supplied with. - We need to train our CHAs more so on commodity management. - HCWs complain IMAM commodities workload is demanding. - HCWs say IMAM commodities is nutritionist work. - Robbery of nutrition commodities from outside. - Beneficiaries sharing commodities with other people which makes them last long. - Shortage of commodities. - Constant transfer of health workers who are trained. - Supply of short expiry date commodities. - Shortage of nutritionists. - Lack of means of transport to do facility to facility commodity transfer. | 1. **Capacity and training gaps**:    1. Lack of tools to help CHPs understand how to use specific commodities.    2. Need for additional training for CHAs on commodity management.    3. Shortage of nutritionists to handle IMAM commodities.    4. Constant transfer of trained health workers disrupts continuity and capacity. 2. **Procurement and supply chain issues**:    1. Difficulty accessing/procuring commodities due to hierarchical bottlenecks.    2. Lack of quantification systems-commodities are not supplied based on actual need.    3. **Challenging with short expiry dates**: some commodities have short shelf lives, making them hard to absorb fully within facilities.    4. General shortage of nutrition commodities. 3. **Operational and logistical challenges**:    1. No transport means for inter-facility commodity transfers.    2. IMAM workload is seen as burdensome; some HCWs feel it should be handled only by nutritionists. 4. **Misuse and loss of commodities**:    1. Robbery of nutrition commodities from external storage or transit points.    2. Beneficiaries sharing their commodities, affecting intended usage and stock duration. |
| **IMAM training and mentorship** | | |
| 1. **How has the IMAM training and mentorship changed HCWs skills in managing acute malnutrition?** | - Usually, when our HCWs are capacity build in terms of management of malnutrition, it improves the management of nutrition cases, malnutrition cases, reporting cases. It improves though we don’t have data to ascertain that. - There is now ability of level 1 workers to support nutrition through community screening. The reason we do screening is to detect malnutrition at very early stage. - We do monthly screening through the platform of adapted mother to mother support group where the program was supporting us. Through that, we are able to identify people who need support and they are referred to the nearest linked health facility. - They are now knowledgeable as compared to before training. They can now report - They can manage malnutrition cases. Both inpatient and outpatient. - They can now write reports perfectly. - They can do diagnosis and make the correct intervention. - Reduced stockout because of ordering in time. - Wastage of commodities has reduced. - The 2024 survey shows that, before we have been above 30% but since 2024 it has come down to 29.1%. - Increased dietary diversity because of training. - Improved child care. | 1. **Improved knowledge and skills:**     1. HCWs are now knowledgeable and can manage both inpatient and outpatient malnutrition cases.    2. They are able to diagnose correctly and make appropriate interventions.    3. HCWs can now write reports accurately and consistently.    4. Improved ability to manage and report nutrition and malnutrition cases. 2. **Enhanced community-level support**:    1. Level 1 workers can now support nutrition efforts through early community screening.    2. Monthly screenings using mother-to-mother support groups help detect malnutrition early and facilitate referrals. 3. **Operational improvements**:    1. Reduced commodity wastage due to better management and ordering.    2. Stockouts have decreased because of timely procurement. 4. **Health and nutrition outcomes**:    1. Increased dietary diversity in the community.    2. Improved child care practices.    3. Reduction in malnutrition prevalences: Survey data shows a decrease from over 30% to 29.1% in 2024. Attributed to training and interventions. |
| 1. **How have IMAM treatment outcomes changed, if at all?** | - When it comes to outcomes, it does not depend on knowledge only, it is also dependent on reception from the beneficiaries. We are still battling cases of uncured clients basically because of other dynamic issues apart from the capacity of the HCWs, we also have other issues that are beyond HCWs. Issues like poverty, food insecurity that causes sharing of the commodities. So, cures go down. We are still facing challenges. - There is reduction of malnutrition cases because of effort we have put together. The children now under program are very few which means we are working. The CHS and CHPs are working. - Defaulters have reduced because of reduced stock outs. - Yes because of outreaches. Cure rate is high because we can reach many people. - The default rate is high because the community here is nomadic, they keep on moving. - The cure rate has improved because they are receiving the right commodities at the right quantity. | 1. **Reductions in malnutrition cases**:    1. There is a reduction in malnutrition cases, as fewer children are now enrolled in the program-indicating effective community health system and CHP efforts. 2. **Improved cure rates**:    1. Cure rates have improved due to:       1. Availability of right commodities in the right quantities.       2. Expanded outreach services that increase access to care. 3. **Default rate trends**:    1. Defaulters have reduced in some areas due to reduced stockouts.    2. However, default rates remain high in nomadic communities due to their constant movement. 4. **Persistent barriers to cure**:    1. Despite HCW capacity, factors like poverty and food insecurity lead to sharing of commodities, which negatively affect cure rates.    2. There are still uncured clients, highlighting that treatment outcomes are influenced by external socio-economic challenges beyond HCW training. |
| 1. **What additional resources or support that could further improve IMAM service delivery?** | - We have our county nutrition plans, we have annual work plan, we have planned activities. One thing I would want from the partners is adherence to the action plan. We have a plan as a department and we have priority areas, the first recommendation I would give, let them prioritize our priority as a department. By them prioritizing what we feel is a priority for us, would have solved this issue. - Request the program to continue supporting us mostly on outreaches because of hard-to-reach areas. - Continue supporting us on printing of IMAM registers. - More trainings and mentorships on nutrition interventions. * - Increasing number of out reaches. - Continue with mass screening for us to identify those cases early in advance. - We need capacity build the CHPs. They must be trained on nutrition issues. | 1. **Alignment with local priorities**:    1. Partners should adhere to county nutrition action plans and department priorities-support should align with what the local department identifies as urgent. 2. **Support for outreach services**:    1. Continued and increased support for health and nutrition outreaches, especially in hard-to-reach areas.    2. Expansion of mass screening efforts to identify malnutrition cases early. 3. **Training and capacity building**:    1. More training and mentorship of HCWs on nutrition interventions.    2. Capacity building for CHPs, particularly on nutrition-related topics.      1. **Tools and materials**:    1. Continued support in printing IMAM registers to facilitate accurate data tracking and reporting. |
| **Integrated Health and Nutrition Outreach** | | |
| 1. **In your view, how have health and nutrition outreach helped increase access to services in remote or underserved areas of Samburu County?** | - They help us to reach the hard-to-reach population because we still have… this county is vast. We still have a population that lives in hard-to-reach areas. ** - It has improved access to health services and nutrition by population who are hard-to-reach. - We can reach the very far end where the government cannot reach to give the services. - We are able to target the malnourished at the hard-to-reach areas. - People don’t have to move many kilometers looking for health services. | 1. **Improved access to hard-to-reach populations** in vast and remote areas of the county. 2. **Extended health and nutrition services** to locations where government services are not easily accessible. 3. Enabled **targeting of malnourished individuals** specifically in hard-to-reach areas. 4. **Reduced the need for long-distance travel** by community members to access basic health services. |
| 1. **Can you provide examples of specific health or nutrition outcomes that have changed due to the outreach sessions?** | - I am not very sure because I didn’t get any data from the outreaches. I might not be able to tell because basically its about immunization and sometimes a bit of nutrition. - If you look at the data within the facility, one of the outcomes is, people with illness that are supposed to be treated in facility are treated at the outreach therefore they are saving them the time and cost of accessing facilities. - When you go to these integrated outreaches, there are many other things that happen. Mothers can access immunization services and you can have nutrition information. For me, doing integrated outreach to help nutrition has been impactful. - There are those mothers who cannot walk many kilometers(60km) to look for health services because they have just come from delivery. Those outreaches really help those mothers a lot. - Children now get full immunization. - Water sanitation has improved because they treat water with help of public health officers. - It has improved linkage between health facility and the community. - Improvement in weight gain. - Communities are embracing vegetable uptake because of BFCI training. | 1. **Improved access to treatment**: people with illnesses that would require treatment at the health facility are now treated at the outreach, saving them time and cost. 2. **Access to immunization and nutrition information**: integrated outreaches have provided immunization services and nutrition information, benefiting mothers and children. 3. **Support for new mothers**: outreaches help mothers who cannot travel long distances e.g., 60km after delivery, offering vital health services at their doorstep. 4. **Full immunization for children**: there is an increase in children receiving full immunization due too outreach programs. 5. **Improved water sanitation**: water is treated with the help of public health officers, improving water quality and sanitation. 6. **Enhanced health facility-community linkage**: the outreach programs have strengthened the connection between health facilities and the communities they serve. 7. **Improvement in weight gain**: there has been an improvement in weight gain, likely related to nutrition interventions at the outreaches. 8. **Increased vegetable uptake**: communities are embracing vegetable consumption as a result of the BFCI training. |
| 1. **What challenges do you face in organizing or sustaining the outreach sessions?** | - Poor coordination because partner should involve the right person to be able to plan for those activities. They should come to the county offices and plan it. - Lack of feedback on how the outreaches are going on. They do it in emails instead of coming to the office to share the feedback. - The sustainability of the outreaches. They are very expensive to maintain. Sometimes when partner pulls out, we have clients who become defaulters. (immunization defaulters, nutritional defaulters…). ** - Another challenge we have is the moving population. You will find them here today but tomorrow they will have relocated. - The county cannot afford the outreaches because they are very expensive. - In the county referral, we need a big storage for the commodities. We need a storage like a warehouse. - Nutrition commodities are not enough. - Poor security, there are some places where you cannot reach. - Constant migration of communities. - Lack of means of transport. | 1. **Poor coordination**: partners fail to involve the right people in planning activities, which affects the overall coordination of the outreaches. 2. **Lack of feedback**: feedback on the outreaches is shared via emails rather than in-person meetings, making it harder to assess the progress and effectiveness. 3. **Sustainability issues**: outreaches are expensive to maintain, and when a partner pulls out, it often results in clients becoming defaulters e.g., immunization or nutrition defaulters. 4. **Moving population:** the migratory nature of the population presents a challenge, as people may relocate before completing their outreach services. Ongoing migration of communities creates challenges in providing consistent and sustainable outreach services. 5. **Limited county resources**: the county struggles to afford the costs of the outreaches, as they are very expensive. 6. **Lack of storage space**: the county referral system lacks adequate storage e.g., warehouses for commodities needed for the outreaches. 7. **Insufficient nutrition commodities**: there is a shortage of nutrition commodities required to effectively run the outreach programs. 8. **Insecurity**: some areas are unsafe, making it difficult to reach certain populations with the outreach services. 9. **Lack of transport**: there is a lack of reliable means of transport, which hinders the ability to conduct outreaches effectively. |

| **Reprinting and use of IMAM registers** | | |
| --- | --- | --- |
| 1. **How has the reprinting and distribution of IMAM registers influenced data quality and reporting on malnutrition cases?** | - It helped in terms of documentation because when you have the right tools, documentation improves. We were able to capture data and when data is captured at the primary level, the quality of the reports is strengthened. - It has improved reference, you can refer - Improved in reporting trends - The data is important because it can be used in decision making in matters of interventions. - The moment we were provided with registers, we were able to report on time. - We can now give accurate data and documentation. - Because of reporting tools, the data comes in time. | 1. **Improved documentation**: availability of the right tools has enhanced documentation at the primary level, strengthening overall data quality. 2. **Better data capture**: accurate data is now being captured at the source, leading to more reliable reporting. 3. **Enhanced referencing**: the registers have improved the ability to reference previous data for follow-up analysis. 4. **Improved trend reporting**: availability of registers has allowed for better tracking and reporting of malnutrition trends over time. 5. **Timely reporting**: the presence of proper tools has enabled timely submission of reports. 6. **Informed-decision making**: the improved data quality supports evidence-based decision making for nutrition interventions. |
| 1. **Are HCWs better equipped to track patient outcomes and treatment data due to the availability of these registers?** | - Apart from IMAM registers, we still require other tools like the reporting tools and we still have a challenge in terms of reporting tools. I would say they are not well equipped. - I would say yes. Apart from registers, the program supported us with MOH100, 515,516… we were struggling with tools for CHPs to capture information and data because they are very expensive. They managed to close that gap by printing tools for level 1 hence improving reporting and reference. Every year we used to have around 2 million budgets for tools which sometimes we don’t have in our budget. So, the program supporting with tools, really equipped HCWs. - Yes, because HCWs were able to trace the defaulters - From the registers, they are able to calculate cure rates and relapse. - The registers help you to know where malnutrition persists. - Yes, those CHPs who have passion do great job of tracking. | 1. **Not fully equipped without complimentary tools**: while IMAM registers are helpful, there is still a challenge due to the lack of other necessary reporting tools, indicating HCWs are not yet fully equipped. 2. **Significant improvement with additional tool support**: The program’s provision of tools such as MOH 100, 515, and 516 helped close gaps, especially for CHPs, improving data capture, reporting, and reference, thereby better equipping HCWs. 3. **Improved ability to trace defaulters**: HCWs can now track patients who miss follow ups, improving continuity of care. 4. **Ability to calculate treatment outcomes**: with registers, HCWs are able to calculate key indicators like cure rates and relapse. 5. **Identification of persistent malnutrition**: registers help HCWs identify areas where malnutrition continues to be problem. 6. **Effective tracking by motivated CHPs**: passionate CHPs use the tools effectively to track patient outcomes and data.   While gaps remain, the availability of IMAM registers and other reporting tools has significantly enhanced HCWs capacity to track and report treatment data and outcomes. |
| 1. **What, if any, challenges remain in maintaining accurate and consistent records across facilities?** | - They are not well equipped because even the registers that were printed , they were not full registers because we require those with hard cover. So, they were used temporarily. We still needed the ideal registers. - We don’t have enough human resource. Like for nutrition, we rely on other carders to help us run our program. - We have challenge with MOH100 tool, we don’t have enough of them. - We have a problem with ECHS system that is currently being deployed to be used in the CU level. Many CHPs and CHAs have not understood it, how to navigate through the system. Which is used to report on service delivery and administration. - Because now we are moving to ECHS, we don’t have the ICT infrastructure in level 2 facilities. - Shortage of staff. We have very few nutritionists, we request county government to employ. * - We still have nurses who don’t know how to report, fill in registers. They need mentorship. - Transfers of the nurses also affects because trained nurses are replaced with those who have not received any training. * - Poor data quality because there are no enough nutritionists in the facility and high workload for the nurses. - Due to high nurse workload, there is inconsistent recording because nurses delegate nutrition (even distributing) to CHPs to do, | 1. **Inadequate and temporary registers**: the registers provided by the program were not durable or ideal e.g., lacked hard covers, making them unsuitable for long-term record-keeping. 2. **Human resource challenges**: according to most county government staff, there are not enough trained personnel, especially nutritionists, leading to over-reliance on other cadres and inconsistent data entry. Some nurses lack the skills to properly report and fill in registers, indicating a need for ongoing mentorship and training. Frequent transfers result in trained staff being replaced by untrained ones, disrupting consistent data recording.    1. “*Shortage of stuff. We have very few nutritionists, we request county government to employ*” **County Government Staff** 3. **Insufficient reporting tools**: there is a shortage of key tools like the MoH 100, hindering consistent data documentation. 4. **ICT infrastructure challenges**: the ECHIS system being rolled out at the community level is not well understood by CHPs and CHAs, making it difficult to use effectively for reporting. Many level 2 facilities lack the necessary ICT infrastructure to support digital record-keeping systems like ECHIS. 5. **Poor data quality**: this stems from both a shortage of nutritionists and the high workload on nurses, which compromises attention to data quality. Due to nurse workload, tasks like nutrition service delivery and documentation are often delegated to CHPs, which can lead to inaccuracies if CHPs are not properly trained. |
| **CHUSLA training for CHPs** | | |
| 1. **How has the training on CHUSLA influenced CHPs ability to generate income?** | - I have not heard anything about CHUSLA - Training CHPS was very influential and momentous because some CHPs have table banking, others are doing other businesses and have become members of some community organizations using the skills of from CHUSLA to sustain themselves. - Mothers have become independent because they have their own groups to make savings. - I am not aware of CHUSLA training. * | 1. **Empowered CHPs through income-generating activities**: training was influential, enabling CHPs to engage in table banking and other small businesses. |
| 1. **Please explain how CHPs are using CHUSLA to generate income?** | - CHPs have done some savings which now helps them start other businesses. Some reported sometime they had saved up to 1 million. They have their time table, they have how you should take a loan and repay it and also have interest rates and regulations. - Yes, because CHPs have their own groups which they make savings. Some have started other businesses as a result of taking loans from those saving groups. - They got knowledge on how to save. | 1. **CHPs are saving and starting businesses**: some CHPs have accumulated significant savings and used those funds to launch income-generating activities. 2. **Functioning savings groups**: CHPs operate organized savings groups with structures systems including timetables, loan terms, interest rates, and regulations. CHPs are taking loans from their savings groups to start new businesses. |
| 1. **How has CHUSLA influenced the financial resilience of CHPs?** | - CHPs are organized into groups apart from doing healthcare services, they are able to loan themselves, they are able to pay their penalties, and they are able to influence the households they have. - Now CHPs have money, they not only depend on the stipend they get from the government but also from other businesses they have started as a result of CHUSLA savings. | 1. **Improved financial literacy**: CHPs gained knowledge on how to save, which supports their ability to manage and grow income. 2. **Enhanced financial independence**: CHPs no longer rely solely on government stipends but also earn income from businesses started through CHUSLA savings. 3. **Access to internal loans**: being organized into groups allows CHPs to loan themselves money, improving their ability to manage financial needs. 4. **Positive influence on households**: the financial stability gained through CHUSLA enables CHPs to influence and possible support the households they serve. |
| 1. **What challenges, if any, exist with CHUSLA?** | - There were areas we were not able to cover. Samburu county is a vast county and the program for some reason could not cover 100%. - Men also need to be involved because most are women. - Also, men should let their women attend the CHUSLA meetings. They should be sensitized about it. | 1. **Cultural or social barriers**: some men restrict women from attending CHUSLA meetings, indicating a need for community sensitization and male engagement. |
| **Closing questions** | | |
| 1. **Is there anything else you would like to add about the program, especially CHS activities impact or areas for improvement**? | - We have a gap with MOH100. We require more MOH100 tools, which are used to refer cases. - We need trainings, sensitization and mentorship to CHPs to be able to understand ECHS (Electronic Community Health System) platform. - Finalizing on the program activities which were left out more on Maralal and Wamba east wards because it was assumed its urban area but we still have population who have gaps. - There is also a gap… we need to implement the revised and renamed module 10 and other modules that have been renamed because there is enhancement of information. - The area of supervision, we still have a gap with our CHSs. Looking at those units in terms of support they require particularly to supervision, we still have a gap. If we can be supported to do the vertical supervision across the county so that we can community healthy units receiving the same attention like any other. But the way it was implemented previously, we were narrowing down to the pockets of the county and leaving out the others. - They should help the CHUSLA members to open bank accounts for saving instead of using boxes to save. It’s not safe. - More trainings on IMAM - We need more reporting tools especially MOH 713. - We need water heaters for inpatient facilities. We only have one in pediatric ward. - When there is training, they should involve health care workers. | |
