# Supplementary material for: Process evaluation of enhanced community health system activities to improve detection and management of acute malnutrition in Samburu County, Kenya
Source: PLOS Glob Public Health. 2026 Mar 24;6(3):e0006010. doi: 10.1371/journal.pgph.0006010 (PMC13012446; doi:10.1371/journal.pgph.0006010)
Supplement: S2 Table — (DOCX) [file pgph.0006010.s002.docx]

**S2 Table. Results and Summaries of In-depth Interviews with Community Health Assistants and Community Health Promoters**

| **Questions** | **Responses** | **Summaries** |
| --- | --- | --- |
| **Mentorship and training on commodity management** | | |
| 1. **How has the training and mentorship on commodity management influenced your ability to manage and provide health and nutrition supplies effectively?** | - It helped because now I have the kits which help in doing first aid and also painkillers which help relieve pain before referring. - There is also dewormers which I give to children. Also, you can do temperature checks because you cannot refer without checking the temperature, so the kit is very crucial. - It has helped us also to single out those malnourished children in the households and referring them to health centers. - It has helped me to know how I can handle a malnourished child as well as counselling the mother because some are adamant to take their children for testing. - I learned that, those supplements are not good for people who are healthy. They should only be used by those who suffer malnutrition, only those in the program. - I have not received training on commodity management. ***** - It has helped me to know how to give out those commodities based on age and weight. * - It has helped to know how to record when giving out the commodities. - The boxes have helped to keep the commodities safe - They showed us how we identify expiry date of those commodities. - We make sure the place we usually store is safe because of rodents. - We also know the quantity a child should receive according to the age. - I always ensure those children in program receive the commodities at the right time and the correct quantity. - For children enrolled in the program who cannot access a health facility-and whose families also face challenges accessing it-I always ensure they receive the necessary commodities, often by delivering them myself. - I always make everything transparent even when issuing those commodities. - We conduct MUAC testing, and if child is identified within the yellow or red categories are provided with the appropriate nutritional supplements. - Since the introduction of commodities, so many people have benefitted including women and children. - Previously, when Plumpy’Nut was distributed, it was meant for children who had been diagnosed with malnutrition. However, some parents would also give it to healthy children. Because of training we received, we had to step in and raise awareness in the community, explaining that giving Plumpy’Nut to children who don’t need it can be harmful. - Before training, we didn’t know what commodity management was, but after training I have gained knowledge on how to handle those commodities and implementing (giving the correct ratio) ** - I am not directly involved in commodity management. I refer clients to the hospital for such services - We have trained CHPs how to use the commodities at the household level. - They have given them smart phones, they can report accurately. - They can trace people at household level and refer them to hospital. - Because of training, we educate community on the importance of these commodities. - Commodity management has helped because there is minimal misuse of the commodities. - It has helped because we now know the levels, we must have for us to request for supply. - It has also helped us to gain knowledge on how we should supply for all beneficiaries in case we have few commodities. - We have bill cards which we record when issuing commodities to CHPs. | 1. **Improved knowledge and skills in commodity management**    1. Most CHPs and CHAs gained understanding of how to give out commodities based on age and weight.       1. “*It has helped me to know how to give out those commodities based on age and weight*.” **CHP**    2. Learned how to record and track distribution of commodities.    3. Acquired skills on identifying expiry dates and proper storage conditions e.g., keeping items safe from rodents.    4. Most CHPs and CHAs are now aware of correct usage and dosage of supplements like Plumpy’Nut to avoid misuse.       1. *“Before training, we didn’t know what commodity management was, but after training I have gained knowledge on how to handle those commodities and implementing (giving the correct ratio)”* ***CHP***    5. Understood that supplements should only be used by malnourished individuals, not healthy ones.       1. “*I learned that, those supplements are not good for people who are healthy. They should only be used by those who suffer malnutrition, only those in the program*.” **CHP**    6. Enhanced ability to calculate and manage supply levels, ensuring timely replenishment.    7. Gained knowledge on how to supply equitably, especially when commodities are limited. 2. **Strengthened service delivery**    1. Can now conduct MUAC testing to identify malnourished children and provide appropriate supplements.    2. Use of kits enables basic health services e.g. first aid, deworming, temperature checks before referral.       1. “*It helped because now I have the kits which help in doing first aid and also painkillers which help relieve pain before referring*.” **CHP**    3. Ensure timely and appropriate distribution of commodities to children in the program.    4. Able to deliver commodities to hard-to-reach families, ensuring no one is left behind.    5. Promote transparency in commodity issuance. 3. **Enhanced community engagement and awareness**    1. Use mentorship and training to educate the community on the importance and proper use of nutrition commodities.    2. Raise awareness on misuse, particularly around inappropriate distribution to healthy children.    3. Provide counseling to caregivers on proper practices and encourage compliance with testing and treatment. 4. **Better monitoring and reporting**     1. Use of smartphones has improved accuracy in reporting and household-level tracking.    2. Equipped CHPs to trace and refer clients efficiently.    3. Implementation of bill cards for commodity issuance tracking to CHPs.       1. “*We have bill cards which we record when issuing commodities to CHPs*” **CHP**   **Challenges noted**   - Most CHAs and CHPs still lack direct training in commodity management. |
| 1. **Please share specific examples of how this training has changed the way you handle commodity shortages or supply issues.** | - We only do referrals, we don’t deal with supply, the nutritionist does the supply and knows how to deal with commodity shortages. - It has helped us realize even when we lack those supplements, we can advise mothers to have a small farm where she can grow different vegetables which can cover over-reliance of supplements which sometimes they might not be there. - By recording, I can track the shortages I have, I contact CHA and there I will have report on what to do. - We have not had shortages but we have facilities close to us which can boost us because we usually work together. - When we have shortages, we always consider those with severe cases first and when we get enough supply, we consider everybody even those who are almost recovering. *** - Additionally, during times of shortages, we teach parents how to prepare various nutritious foods that can serve as alternatives to the provided commodities. ** - We now request the commodities on time, before we could do it after its depleted. - Educate mothers and caregivers on how to reduce malnutrition through balanced diets. - Encourage men to support their wives in ensuring proper nutrition at home - When we have shortages, we reduce the number of commodities we give out. For example, we can just give out two Plumpy’Nuts per beneficiary, then advise them to come next week. - We give commodities to CHPs based on the number of households they have. - We report in time so that we don’t go out of stock. | 1. **Improved planning and timely requesting**     1. Now request commodities on time, preventing total depletion before restocking.    2. Report stock levels in time to avoid running out.    3. Track shortages through records and communicate with CHAs for necessary action. 2. **Prioritization during shortages**     1. Most CHAs and CHPs in shortage situations, prioritize severely malnourished cases first, then support recovering cases when more supplies arrive.       1. “*When we have shortages, we always consider those with severe cases first and when we get enough supply, we consider everybody even those who are almost recovering*” CHP    2. Adjust rationing e.g., reduce quantity per beneficiary e.g., two Plumpy’Nut per child and advise return visits. 3. **Use of alternative nutrition strategies**     1. Educate caregivers on home-based alternatives, like growing vegetables to reduce reliance on supplements.       1. “*We advise mothers to have a small farm where she can grow different vegetables which can cover over-reliance of supplements which sometimes they might not be there*” **CHP**    2. Most CHAs and CHPs during shortages, teach parents how to prepare nutritious local foods as substitutes.       1. “*During times of shortages, we teach parents how to prepare various nutritious foods that can serve as alternatives to the provided commodities*” **CHP**    3. Promote balanced diets and nutrition education for caregivers.    4. Involve men in household nutrition, encouraging them to support proper feeding practices. 4. **Enhanced community and facility collaboration**    1. Leverage support from nearby facilities when shortages occur, through collaboration and mutual aid.    2. Allocate commodities based on household numbers per CHP, ensuring fair distribution. |
| 1. **What challenges do you still face in managing these commodities?** | - After referring a child to health center, you don’t know whether he/she will get help. You will not know whether he/she will be introduced to “Uji” and when they will end it. - Long distance between households and facility which makes most miss the commodities. They just ignore even after requesting them to come and pick. - In our facility, we don’t have ambulance. - People like free things, in the process of giving out the supplement, we give based on condition. Some people will start to hate you because they didn’t receive which is not our fault, we give priority to the most affected first. - Theft of the commodities. - Sometimes there is shortage of commodities despite high demand from the community. - Some commodities are not enough. You may find people with injuries and you don’t have enough gloves to handle that. - Due to long distance and lack of means of transport, I can’t carry all the commodities when visiting them. - People defaulting on using those commodities. - Some share plumpy’nut. In one case after realizing that. The child had to start taking the plumpy’nut from the clinic and then goes home on daily basis. - Some parents were sharing Plumpy’Nut with other children who were not affected. We explained to them that doing so could lead to infections, which helped reduce this behavior. ** - Some were chewing Miraa with Plumpy’Nut. - Some don’t know the importance of Plumpy’Nut, so you have to sit down and advice the person. - Because our people love free things, they want the Plumpy’Nut even when they don’t have any affected children. - Some of the affected people we give Plumpy’Nut they end up selling them at Ksh20. * - Some community members blame CHPs for not directly bringing health supplies to them. - Shortage of commodities. - Lack of knowledge on commodities from the CHPs. - When CHPs issue out commodities, they don’t come for another supply or they come back late. - When we request all CHPs to come for the commodities, not all who turn up. If you have around 10 CHPS, only lie 5 will turn up. - We lack storage for the commodities in our dispensary which forces us to store them with other drugs. - Because of illiteracy, many community members don’t know much on commodities and their uses. | **However, despite the observed changes and success factors, there are still challenges on:**   1. **Logistics and accessibility challenges**     1. Long distances between households and facilities limit access to commodities.       1. “*Long distance between households and facility which makes most miss the commodities. They just ignore even after requesting them to come and pick*.” **CHP**    2. Lack of transport/ambulances affect referral and delivery of commodities.    3. CHPs sometimes cannot carry all commodities when visiting households due to distance or lack of means.    4. According to CHAs, low turnout of CHPs when called to collect supplies for example only a fraction turn-up.    5. According to CHAs, CHPs delay in retuning for resupply, disrupting distribution continuity. 2. **Supply and storage issues**    1. Frequent shortages of essential commodities despite high community demand.    2. Inadequate quantity of supplies e.g., gloves, supplements to meet demands.       1. “*Some commodities are not enough. You may find people with injuries and you don’t have enough gloves to handle that*.” **CHP**    3. Lack of proper storage facilities, forcing commodities to be stored with other drugs, which can lead to mix-ups or spoilage.    4. Theft of commodities is a risk, compromising availability. 3. **Misuse and misunderstanding of commodities**    1. According to most CHPs, Plumpy’Nuts are being shared among children, even those not malnourished.       1. “*Some parents were sharing Plumpy’Nut with other children who were not affected. We explained to them that doing so could lead to infections, which helped reduce this behavior*.” **CHP**    2. Some beneficiaries sell Plumpy’Nut e.g., at Ksh20, defeating its purpose.    3. Use of Plumpy’Nut alongside harmful substances like Miraa.       1. “*Some were chewing Miraa with Plumpy’Nut*.” **CHP**    4. Some community members do not know the importance of the supplements, requiring ongoing sensitization.    5. Illiteracy and general lack of awareness among community members lead to misuse or underuse of commodities.    6. Defaulting on commodity use. Not completing or following dosage instructions.      1. **Social and community pressure**     1. Some community members blame CHPs for not delivering supplies directly to their homes.    2. Perception by some community members that CHPs favor some people due to giving based on condition. This leads to resentment or conflict.    3. Some people demand free commodities even when they are not eligible, due to a culture of expecting handouts. 2. **Monitoring and referral gaps**     1. After referral, some CHAs do not receive feedback. They do not know if a child received help or what was prescribed. 3. **Knowledge and capacity constraints**     1. Some CHPs still lack adequate knowledge about commodity use and management.    2. Community-level knowledge gaps hinder proper usage and support of nutrition interventions. |
| **IMAM training and mentorship** | | |
| 1. **How has the IMAM training and mentorship affected your skills in identifying and managing malnutrition cases?** | - I have not done training on IMAM. ** - I can take measurements on weight, height and MUAC and if the child is malnourished, I refer. **** - If the child is found to be malnourished, will be put under program and after there I will be making follow ups. - It has helped me to identify children who are malnourished because I couldn’t do that before. **** - Now, I can screen KGs, height and temperature of a child before referring them. - I can give them Vitamin A and deworming. - I have not received training on IMAM - At least now we are in a position to screen and identify those cases of malnutrition in our community and refer them. - We also know the number of commodities one should receive per day. - It has upscaled my skills. Through training, we trained on management of moderate and severe, malnutrition among the infant which has enabled me to train CHPs. - Because of training, I have been also able to train CHPs on MUAC. - After screening, if child is malnourished, I will refer and from there I will be monitoring to make sure they get the commodities until they get well. * - For malnourished children, we screen for MUAC, height and weight. We assess to see those who can be enrolled to the program. * - For pregnant women, we usually advise them to start clinics immediately after they test positive for pregnancy because many cases of infant malnutrition are as a result of failing to attend clinics. - We conduct MUAC testing, and if child is identified within the yellow or red categories are provided with the appropriate nutritional supplements. After two weeks, the child is reassessed to determine any signs of improvements. - I always advise women to have small farms like kitchen garden where they can grow vegetables because they will be nutritious to the children and malnutrition will greatly reduce. - I also advise them to consider giving their children fruits because they also help in nutrition. - It has helped us because we can manage malnutrition cases at household level before reaching healthy facility by creating awareness. - Also, at household level we could screen and if they are malnourished, we indulge them into the program. - It has improved my skills in identifying malnutrition cases using anthropometric measurements - It has sharpened my skills because even at household level I can test MUAC for my kids and my wife. - I got skills in management of acute malnutrition on transferring those children who are on OTP to SSD and how to cure them. - If you test MUAC, children under the red MUAC you refer. Now, doctor will also test to affirm that. If the results remain the same, the child will be put under supplements. | 1. **Enhanced skills in identification of malnutrition**     1. According to most CHPs, training has improved their ability to screen for malnutrition using MUAC, height, and weight measurements.       1. “*I can take measurements on weight, height and MUAC and if the child is malnourished, I refer*.” **CHP**    2. Most CHPs could not identify malnourished children before, but now can confidently detect and refer cases.       1. “*It has helped me to identify children who are malnourished because I couldn’t do that before*” **CHP**    3. CHPs can now screen children at household level before referral.    4. CHPs are able to identify malnutrition categories e.g., yellow, red on MUAC tapes and initiate appropriate follow-up.    5. Some community members can even screen their own family members. 2. **Improved referral and follow-up practices**    1. After identifying malnutrition, most CHA/CHPs now refer children to health facilities and continue to monitor their progress.       1. “*After screening, if child is malnourished, I will refer and from there I will be monitoring to make sure they get the commodities until they get well*.” **CHP**    2. Some CHAs follow up to ensure children receive the correct commodities and continue treatment until recovery.    3. Most CHPs understand the enrollment process into nutrition programs after identification.    4. Some CHPs support community education on nutrition, creating awareness on when and why to seek help. 3. **Capacity to manage malnutrition at the community level**     1. Most CHPs can now manage some malnutrition cases at household level through awareness and support before escalation.    2. Some CHPs advise families on nutrition practices e.g., growing kitchen gardens, feeding fruits to prevent malnutrition.    3. CHAs have learned how to manage moderate and severe malnutrition, including how to transfer children between treatment phases for example OTP to SSD. |
| 1. **What changes in treatment outcomes (e.g., cure rates, reduced relapse), if any, have you noticed since receiving this training?** | - I am not aware of IMAM - There is a lot of improvement because earlier you could find people have been under program for long time but now even some can take one month because of the trainings we give them on alternatives if we don’t have supplements. - For example, we have trained them on chicken rearing and in return they can get eggs which is very nutritious for children as well as adults. Those are some of the alternatives. - Also, the number of malnourished children has gone down since many of those affected have been put under the program. - There is a lot of improvement because they get quality health services immediately. Because of this, cases of malnutrition have significantly reduced compared to before. - There is improvement because when we do screening, we detect this malnutrition early and refer them to health centers and get the commodities at the right time and the disease is managed before it becomes severe. - There is also improvement because we advise them on how to eat balanced diet - There is also food security whereby the program came in and supported mother to mother support group through inputs such as seeds for planting. - It has helped mothers to know how to cook. - There is improvement because there are no many cases of severe malnutrition because they can be detected early. - It has improved because we now know the ratio a child is supposed to take in relation with weight and MUAC. - Those I have referred, received good treatment and now are very healthy. - When training was done, the number of children with malnutrition in the health facility increased because we went around looking for them because they were being hidden at home before. - There is improvement because since we sensitized them on vaccination, the number of those receiving vaccination has increased. - The cases of malnutrition have gone down because we trained parents on nutrition. - There was a child who was severely malnourished and after testing MUAC was in the red zone, we gave appropriate commodities and after two weeks the child recovered. - Because of increased uptake of vegetables, the malnutrition cases have reduced. - There is big improvement because many cases which we were handling at healthy facility level, we are doing them at household level hence reaching more who are malnourished. - Yes, I can identify cases early, refer them for treatment, and manage follow-up - There has been a lot of improvement because we have sensitized the community on how to prevent malnutrition by eating vegetables and other foods. - At least now community members know much about diet. - Last year, we had many malnourished children but after giving them commodities, most of them got healed. - There are those who default but through CHPs we get them back to the program. - There is improvement because if a child is put under a program and maybe the period is supposed to be under program is over and yet to improve, is taken back to the program. - There is reduction of malnutrition cases. - Because of follow ups, there is big improvements. | 1. **Improved cure rates and recovery time**     1. **Faster recovery times**: previously, individuals remained in the program for long periods, but now some recover within a month, aided by nutrition education and alternative solutions.    2. Specific example: a severely malnourished child recovered within two weeks after being put on appropriate commodities and follow-up.    3. Many children who received commodities recovered and are now healthy. 2. **Reduction in malnutrition and relapse cases**     1. Number of malnourished children has significantly decreased due to earlier detection and consistent treatment.    2. Severe malnutrition cases have reduced, attributed to early screening and prompt referral.    3. Fewer cases of relapse, as CHPs follow up and return defaulters to the program for continues treatment.    4. Children who do not recover within the expected time are reintegrated into the program, ensuring continued care. 3. **Early detection and preventive care**    1. Improved screening skills allow for early detection, leading to early treatment before conditions worsen.    2. Increased awareness in the community has helped bring hidden cases to light, boosting treatment numbers and reducing overlooked malnutrition.    3. Many cases are now handled at household level, expanding reach and reducing facility overload. 4. **Enhanced community nutrition education**     1. Training parents and caregivers on nutrition, kitchen gardening, and balanced diets has improved household dietary practices.    2. Programs encouraging chicken rearing and vegetable consumption have improved household food diversity and reduced malnutrition incidence.    3. Sensitization on vaccination and maternal health has contributed to better child health outcomes and lower rates of malnutrition. 5. **Strengthened health system linkages**     1. Timely access to quality health services has improved outcomes, especially when malnourished children are referred early and commodities are available.    2. CHPs and CHAs are now more equipped to track dosage ratios based on MUAC and weight, improving treatment precision. |
| 1. **What additional support, if any, would you need to implement IMAM services?** | - I am not aware of IMAM - More trainings to CHPs on IMAM will greatly help********* - More tools i.e. MUAC, weight tool, Height tool. - Support on dialogue days so that when we gather community, we can share the information, - If we can get more commodities because sometimes you have more children than the commodities. - Capacity building on community health workers so that they know how to handle the commodities. - Training on commodity management - Provision of more tools like weight scale and height board. - More supplements on health centers. - Refresher training on IMAM. *** - Training of pregnant mothers and those with children under 5 years old. - Electronic system that will help in managing malnutrition cases. A system that can store information. - Training CHPs and their households, including on sensitive topics like SGBV (sexual and gender-based violence) - Continued community support and CHPs stipends - Training of CHAs and HCWs. - Provision of family led MUAK to every household. - Training community members and CHPs. - Screening support in order to do mother to mother screening every month. - They support us to reach hard-to-reach areas because those are the areas where malnutrition cases are high and also, they don’t come to facilities because its very far. - I request support in mass training because through that is when you will know how your community is doing. - Support in out reaches because its only that we can reach hard-to-reach population. - Support in spread of health messages at the community level. - Making commodities available at every facility. - Facilitation support to CHPs to motivate them. | 1. **More capacity building and training**    1. According to most CHPs they need more trainings for CHPs on IMAM to build knowledge and improve case management.    2. Refresher training on IMAM to update and reinforce skills.    3. Training on commodity management to improve handling and distribution of nutrition supplies.    4. Training of pregnant mothers and caregivers of children under 5 years on malnutrition prevention and care.    5. Capacity building of CHPs to ensure competent management of malnutrition cases.    6. Training community members to promote early identification of malnutrition.    7. Incorporate sensitive topics like SGBV in CHP and household-level trainings. 2. **More tools and supplies**     1. Provision of more MUAC tapes, weighing scales, and height boards for accurate screening.    2. More nutrition commodities and supplements, especially during shortages or high demand.    3. Making commodities consistently available at all health facilities.    4. More family-led MUAC tapes for each household to support early detection at home. 3. **More community engagement support**     1. Support for dialogue days to enable community gatherings and information sharing.    2. Support for outreach programs to reach hard-to-reach and underserved populations.    3. Screening support for regular community-based checks e.g., mother-to-mother monthly screening.    4. Support in spreading health messages at the community level to increase awareness and knowledge.    5. Mass training support to assess community health knowledge and mobilize collective action. 4. **Improve systems and infrastructure**     1. Electronic systems for malnutrition case management, including data storage and tracking tools.    2. Consistent facilitation and stipends for CHPs to keep them motivated and reduce attrition.    3. Improved logistics support, especially for remote areas with limited access to health facilities. |
| **Integrated Health and Nutrition Outreach** | | |
| 1. **How have you been involved in integrated health and nutrition outreach, and what role do you play in these events?** | - I was involved in one for nutrition which we were doing demonstrations on how to prepare food and making sure it is balanced. I was testing MUAK among the children, the pregnant women and those women with children under 5 years of age. - I was also sensitizing them on the importance of having toilets. - Sensitizing the community on coming event and how important it will be. * - Coordinating with them during the event by assisting where necessary. - Taking reports from my households when we meet. - Mobilizing households for the event. ********* - Screening of height, weight and MUAK. ****** - Offering health education and why it is important for them to participate. *** - I do screening of pregnant women and children under 5 years old. - Deworming and giving out vitamin A. ** - Arranging the venue - Issuance of health commodities. - Nutrition counselling - Health counselling more on hygiene - Educating my households on importance of family planning and good healthy. - I get an opportunity to tell the community that we work hand on hand with CHPs, with that the work of CHPs becomes easy because there is trust from the community. - Getting to know the problem my household members have before even coming to outreaches. This will help to tailor the services towards those problems. - Those fear to share with the doctor, I usually inform him on their behalf. * - Making follow-ups after the outreach. * - Mobilize the community through CHPs. * - I usually give health talk during those events. - We have not done any outreach in my CU. * | 1. **Community mobilization and engagement**     1. According to most CHPs, mobilizing households and the wider community to attend outreach events.    2. Sensitizing the community about upcoming outreaches and their importance.    3. Building trust between the community and CHPs, making it easier to deliver services.    4. Identifying households-level problems in advance to tailor outreach services effectively. 2. **Screening and health assessments**     1. According to most CHPs and CHAs, screening children under 5 and pregnant women for malnutrition using MUAC, height, and weight measurements.    2. According to most CHPs and CHAs, deworming and administering Vitamin A supplements during the outreach.    3. Monitoring and documenting household health status for follow-up and reporting. 3. **Health and nutrition education**     1. According to most CHAs and CHPs, providing health and nutrition counseling, including:       1. Demonstrations on how to prepare balanced meals.       2. Education on hygiene and sanitation e.g., importance of toilets.       3. Family planning and general wellness.    2. Conducting health talks during outreach events. 4. **Service delivery and logistics**     1. Issuance of health commodities during outreach events.    2. Assisting with event logistics, such as arranging the venue.    3. Supporting coordination during the event by helping with various activities as needed.      1. **Follow-up and reporting**     1. According to most CHPs and CHAs, making follow-ups after the outreach to ensure service continuity.    2. Reporting household health issues to health professionals when individuals are reluctant to speak directly.    3. Taking and sharing household reports during outreach meetings. |
| 1. **In what ways do these outreach sessions help you engage with the community and provide services?** | - It helps because I can reach people who live far away and enable them to access hospital services closer to their homesteads hence breaking distance barrier. **** - It makes my work easier since we can reach many people with health services. - It reduces the challenge of walking long distances, as people can easily get services without travelling far. * - Many people will receive health services with ease of travelling and also will help me collect my household reports with ease because when collecting from household sometimes you miss those individuals that makes you plan a return on another day. - It has helped me because I can screen children on my own. - I can also educate lactating mothers about exclusive breastfeeding. - It helps me because my community can receive health services even the elderly and those children who can’t access health facilities. - It helps build trust and good relationship between the community and myself because of those services. ** - Closes the gap between those who can access health services and those who couldn’t access. - It empowers the community because they receive those health services in their areas. - It promotes immunization - It helps to access those people who do not visit health facilities. - It helps me visit my villages which I couldn’t manage to access because of long distance. - The outreach will help me identify new infections in the community incase for those who hide. - It helps me because I will get reports from there. - The events give me opportunity to so community dialogue which help us plan for action days and also pass crucial information. - Through outreaches, because transport is provided, I can reach out to many clients. - We get opportunity to see even those children who have been hidden maybe because of disability. We get opportunity to advise those families to visit health facilities for further checkups and support. - It brings together my households to receive health services. | 1. **Improved access to services**     1. Help most CHAs and CHPs reach people in remote areas, reducing distance barriers to health facilities.       1. “*It helps because I can reach people who live far away and enable them to access hospital services closer to their homesteads hence breaking distance barrier*” **CHP**    2. Allow community members-especially the elderly and children to access services without traveling long distances.    3. Provide a way to serve those who normally do not visit health facilities.    4. According to most CHPs, it closes the gap between those who can and cannot access health services.    5. Transport provided during outreaches enables CHPs/CHAs to reach more clients. 2. **Increased service coverage**     1. Enable mass screening e.g., children for malnutrition.    2. Promote immunization uptake within the community.    3. Promote health education e.g., exclusive breastfeeding for lactating mothers.    4. Allow CHPs to collect household health reports more effectively than door-to-door visits. 3. **Strengthening community engagement**     1. Build trust and stronger relationships between CHPs/CHAs and the community.    2. Bring households together to receive services, creating a sense of unity and shared responsibility.    3. Support community dialogue, which aids in action planning and spreading vital health information. 4. **Early detection and follow-up**     1. Help CHPs identify hidden or unreported health issues, such as malnutrition or disabilities.    2. Facilitate referrals by spotting new infections or health concerns during outreaches.    3. Enable follow-up with households that may be hard to reach individually. |
| 1. **What challenges or barriers do you encounter in organizing or conducting the outreach sessions?** | - Mobilizing people during market days is very difficult which makes us sometimes shift outreach to the other days. - Long distances *** - Rain disturbances * - If the person isn’t available at the household, you’re forced to make a return visit on another day. - The community believe we benefit from the outreaches while them they don’t get anything financially. They think we use them for financial gains. - Lack of means of transport. *** - Poor network connectivity. It’s hard-to-reach people via phone. *** - Even after mobilizing, some refuse to attend yet they have health issues because of ignorance. *** - Lack of particular medicines for particular diseases. - Poor infrastructure like roads when there are rains. ** - High level of illiteracy from the community about the malnutrition. ** - They demand for money yet we are giving them free services. - There are those who cannot even get out of the house yet they are very sick, more so the elderly. They cannot access where the outreach is. - Since the outreaches stopped, so many children cannot access healthy facility because of long distances. - Because of pastoralism, there are those who don’t attend, they prefer going to herd. - Lack of credit to mobilize, prompting you to move around physically mobilizing. - I don’t face any challenges. - Insecurity *** - Spending a lot of time explaining the community about the commodities. They take time to understand. - We lack anthropometrics like weighing scale, height scale… - You cannot get people unless you inform them days before because they migrate. | 1. **Logistical and Geographical barriers**     1. According to most CHPs, long distances between households and outreach locations.    2. Lack of transport, making it hard to reach remote or scattered households.    3. According to most CHPs, poor road infrastructure. Especially during rainy seasons, which hampers mobility.    4. According to most CHPs, rain disturbances can delay or cancel planned outreach events.    5. Poor network connectivity makes it hard to mobilize people or communicate effectively.    6. Nomadic or pastoralist lifestyle require early notice or make some people consistently unavailable. 2. **Community perceptions and participation challenges**     1. Low turnout on market days requiring rescheduling of outreaches.    2. Some community members believe CHPs benefit financially from the outreaches.    3. According to most CHPs, ignorance or lack of interest in health services causes some to refuse attending, even when unwell.    4. Demands for money or incentives, despite services being free.    5. According to most CHPs, high illiteracy levels leading to difficulty understanding health message or malnutrition.    6. Mistrust or misunderstanding about the purpose of outreaches and commodities.      1. **Service delivery constraints**     1. Lack of specific medicines for certain conditions during outreaches.    2. Insufficient anthropometric tools e.g., MUAC tapes, weighing and height scales.    3. Spending a lot of time explaining commodities and services due to low awareness or misinformation. 2. **Accessibility issues**     1. Some individuals especially the elderly or sick are unable to reach outreach locations.    2. Household members not being present at the time of visit, forcing return trips.    3. According to most CHPs, insecurity in some areas limits the reach and safety of outreach services. |
| **CHUSLA training** | | |
| 1. **What was your experience with the CHUSLA training? How has it helped you in generating income?** | - We have realized we can help ourselves through the little savings that we do. - We share what we have saved through merry go round which helps us to cater for the needs in our households. - It also helps us to meet regularly which builds our relationship among us. - It helps because it has small interests unlike loans. - If your family member falls sick, you can easily access the money through a loan. - Also, helps because you can settle school arrears easily through a loan. ** - Because of the training, we started saving which has impacted our life because at the end of the year you have something which you can buy something for your family. - It has helped because you can take a loan from the savings to boost your business or start a business. **** - The savings can be used as an insurance whenever one of us has a problem (death or sickness), they can receive something from those savings. - Children now can go to school because you can take a loan to pay school fees. ****** - Improved diet because if you take a loan to open a business, the profits you get will help you cater for your family needs and able to eat balanced diet. - It was good experience because we elected chairman, secretary, treasurer, and key holder. - It helps in case you have an emergency. You can take loan from the group. - We have gathered business ideas - It has empowered the community because you can borrow money from those boxes. - Taking loan from the group has helped me to cater for the needs of my family. - The training has helped me to know how to manage my money. - It has helped because we have learned the importance of saving. - It has helped so much because we started in August last year andhave now managed to save Ksh50,000 - It is easy to access loans in those groups instead of going to the bank which is very far. - It has made things easy because I don’t have to travel long distance to access a loan. The banks are very far from here. So, that issue is now solved because I can get a loan form the saving group. - We don’t have to sell our livestock to settle something, you can now take a loan to do so. - There is not a lot of guaranteeing, unlike banks. - It has improved my skills because I trained as a TOT, which helped me train many mother-to-mother support groups. - Participate in the VSLA group, contribute to saving, and access loans for emergency cases. - Now, we have table banking which has enabled us to do farming. - The training has helped me do personal savings but I am not in CHUSLA groups. - I can address concerns which CHPs raise concerning their CHUSLA groups. - Because of CHUSLA training, we now have a welfare. | 1. **Improved financial management and savings culture**     1. CHUSLA training taught the importance of saving, enabling CHPs/CHAs to accumulate funds for personal or family needs.    2. Participants now save regularly, with some groups managing to save up to Ksh. 50,000 since starting.    3. The training has helped them manage money better and build financial discipline. 2. **Access to loans and financial security**     1. Easy access to loans through savings groups has replaced the need to travel far to banks.    2. According to most CHPs, loans are used to:       1. Pay school fees for children.       2. Settle emergencies such as sickness or death in the family.       3. Boost or start businesses.       4. Pay off school arrears       5. Improve household diets through business profits.    3. Loans are low-interest, easily accessible, and don’t require heavy guarantors, unlike banks.       1. “*Helps because you can settle school arrears easily through a loan*” **CHP**      1. **Income-generating activities and empowerment.**     1. Members have started or expanded businesses using group loans.    2. Table banking enabled farming, improving food security.    3. Business ideas are shared and explored within group setting.    4. Some CHPs have become Trainers of Trainers (ToTs), enabling them to train other support groups and spread impact. 2. **Social benefits and support systems**     1. CHUSLA groups serve as informal insurance schemes, offering financial aid during illness or death.    2. Welfare systems have been established through CHUSLA.    3. Merry-go-around contributions support household needs and foster unity.    4. Regular meetings strengthen relationships and community cohesion. |
| 1. **How has CHUSLA affected CHPs ability to generate income?** | - They have trained us on small projects like chicken rearing or kitchen gardens which helps us in boosting our financial income. - By saving and loans, you can start a small business like a kiosk. **** - It has helped me gather business skills. - It has allowed me to begin farming and raising goats through a loan, and after harvesting, I earn profits that help me repay the loan along with interest. - CHPs have farms which they grow vegetables - They have been able to start business like livestock and chicken rearing through loans. ** - Because of CHUSLA, the money we save, people have started businesses like kiosks, hotels and Saloons. - Women have even started keeping livestock because when we share the savings, some buy goats. - They have formed groups for saving. - Some CHPs take loans to start vegetable business which helps them make profits. | 1. **Support for income-generating activities**     1. CHPs have been trained on small projects such as chicken rearing and kitchen gardening to boost household income.       1. “*They have been able to start business like livestock and chicken rearing through loans*” **CHP**    2. CHUSLA has enabled CHPs to start small businesses like Kiosks, hotels, and salons through access to savings and loans.    3. Loans are used to begin farming, raise goats or chickens, and have after harvest/sales, profits are used to repay loans and support families.    4. CHPs have started vegetable farming and livestock keeping (goats and chickens) as viable income sources. 2. **Financial empowerment through group savings**     1. CHPs have formed savings groups where they contribute regularly and access funds to invest in income-generating activities.    2. Some use shared savings to purchase livestock, such as goats, increasing asset ownership and future earning potential.    3. CHUSLA has helped CHPs develop business skills, making them more confident in starting and managing ventures. |
| 1. **Which barriers have you experienced with CHUSLA?** | - Lack of money when it’s time to contribute ****** - The money which we share at the end of the money is not enough to cater for everything**.** - There is no barrier* - You can take a loan to do business but unfortunately the business fails to pick. - CHPs are not willing to attend meetings. - People take loans and they end up defaulting**. **** - Poor recording because of illiteracy. - We lack a box for our savings. ** - The days we usually meet some don’t come because of long distances and they have no phone which you can reach them. - Risk of carrying money with a shopping bag because we don’t have a box. - Lateness to repay the loans. - Delays in contribution because some CHPs are unwilling to contribute. - Ignorance from some members. You can find some going for months without contribution. * - Because of different savings contributions, some contribute more than others, which can make some think of quitting. - Migration of the community to far places really affect continuation of those CHUSLA groups. | 1. **Financial constraints**     1. Lack of money when its time to contribute.    2. The shared amount at the end is often insufficient to meet all needs.    3. Loan defaulters by some members.    4. Lateness to repay the loans.    5. Irregular contributions by some CHPs.    6. Risk of business failure after taking a loan. 2. **Group participation and commitment**    1. Low meeting attendance by CHPs.    2. Delays in contribution because some CHPs are unwilling to contribute.    3. Ignorance or lack of commitment from some members.    4. Migration of the community to far places affect group continuity. 3. **Logistical and infrastructure challenges**     1. Distance and lack of phone access hinder member participation.    2. Lack of a secure saving box.    3. Risk of carrying money in shopping due to no box. 4. **Group management and fairness**     1. Poor financial record-keeping due to illiteracy.    2. Unequal contributions cause tension among members. |
| **Section 4: Closing questions** | | |
| 1. **Is there anything else you would like to add about the program, especially CHS activities impact or areas for improvement?** | - The program has helped in promotion of nutrition among malnutrition and women empowerment. - The supplements they should make sure they reach specifically to those children who are affected because sometimes it does not. - I request more trainings to the community members and CHPs specifically on IMAM ****** - Provision of tools. MUAK, weight tool and height tool. *** - Provision of transport means - Construction of more health facilities - Training of CHPs on community health information system. - Training of commodity management and CHUSLA. *** - They should continue with Nutrition program. - Increase in number of mother-to-mother groups. - They should continue with community outreaches and making sure its frequent. - Training of VSLA - During mobilization, receive a token of appreciation because even water we don’t receive. - Refreshment courses for CHPs. - Follow-up visits should be consistent - The program has helped improve community health. - We need support in father-to-father support groups. - We need training facilitation. - The program has really helped us gain knowledge more so on IMAM. - I would request support with outreaches for hard-to-reach areas. - Monthly token appreciation for CHPs because they do voluntarily. - The program should know areas of jurisdiction because some facilities were left out in the previous program which affected even the entire community. - The trainings to the community and CHPs should happen regularly. - Refresher on CHUSLA training.   Because now we are using electronic system to report (ECHS), most of them don’t have data to synchronize, we need support in that. | |
